# Evaluation of the Effectiveness of Compression Therapy Combined with Exercises Versus Exercises Only Among Lipedema Patients Using Various Outcome Measures

**DOI:** 10.3390/life14111346

**Published:** 2024-10-22

**Authors:** Monika Czerwińska, Marcin Gruszecki, Jacek Rumiński, Rita Hansdorfer-Korzon

**Affiliations:** 1 Department of Physiotherapy, Medical University of Gdansk, 80-211 Gdansk, Poland; rita.hansdorfer-korzon@gumed.edu.pl; 2 Department of Radiology Informatics and Statistics, Faculty of Health Sciences, Medical University of Gdansk, 80-211 Gdansk, Poland; marcin.gruszecki@gumed.edu.pl; 3 Department of Biomedical Engineering, Faculty of Electronics, Telecommunications and Informatics, Gdansk University of Technology, 80-211 Gdansk, Poland; jacrumin@pg.edu.pl

**Keywords:** lipedema, lipoedema, compression therapy, qualitative measures

## Abstract

The treatment of lipedema remains challenging, largely due to widespread misconceptions. Selecting the appropriate treatment method necessitates the use of accurate outcome measures. This study aims to evaluate the effectiveness of compression therapy combined with exercises versus exercises alone in lipedema patients using various outcome measures. Twenty-four women with lipedema were divided into two equal groups: one group received compression therapy plus exercises while the other group performed exercises only. The effectiveness of the treatment was assessed before and after the intervention using several measures: an SF-36 questionnaire, a symptom severity survey, circumference (via 3D scanning), and body composition analysis. Significant improvements were observed in the SF-36 Physical Functioning and SF-36 Energy/Fatigue scores among participants in the compression group. Additionally, there was a reduction in the heaviness of extremities, the disproportion between the trunk and limbs, and the level of swelling in the compression therapy. Circumferences decreased in both groups. Although more circumferences were significantly reduced in the compression group, the reduction at the point above the knee was greater in the non-compression group. Compression therapy is an effective treatment for lipedema. Various measures, such as quality-of-life questionnaires and symptom severity surveys, can be used as valuable tools for assessing the effectiveness of lipedema treatment.

## 1. Introduction

Lipedema can be described as the painful accumulation of subcutaneous adipose tissue around the extremities, leading to a disproportionate body shape [1,2,3,4,5]. Its onset is correlated with hormonal changes during puberty, pregnancy, or menopause, and it occurs mainly in the female population [5,6,7]. Symptoms of lipedema include heaviness in the extremities, pain (both spontaneous and upon palpation), disproportion between the trunk and limbs, and a tendency to bruise easily [3,8,9]. The most characteristic feature of lipedema, essential for diagnosis, is pain in the affected areas [5,10,11]. The intensity of lipedema symptoms varies among individuals and is not necessarily linked to the stage of the disease. These symptoms significantly impact patients’ daily functioning and quality of life [12,13,14].

Despite increasing interest from researchers and clinicians in recent years, lipedema remains underdiagnosed and often overlooked [15,16]. The exact incidence is still unknown, but it is estimated that up to 11% of women may be affected by this condition [17]. The absence of clear and homogenous diagnostic criteria often leads to misdiagnosis, which delays the application of proper management [4,5,18].

Current treatment recommendations for lipedema are divided into conservative and surgical approaches [15,19]. Conservative treatment focuses mainly on reducing lipedema symptoms and improving patient mobility. Commonly suggested methods include compression therapy, exercise, weight management, psychological support, and additional treatments such as shock wave therapy, deep oscillation, and pneumatic compression [19,20]. Previously, all modalities of Complete Decongestive Physiotherapy were recommended. However, the use of Manual Lymphatic Drainage (MLD) in lipedema is now under discussion [5]. In the pure form of lipedema, there is no fluid accumulation as seen in conditions like lymphedema, which means MLD might not effectively reduce limb volume [5]. Numerous previous studies have focused on the effectiveness of combining all components of Complete Decongestive Therapy, including Manual Lymphatic Drainage, compression therapy, Intermittent Pneumatic Compression, and exercise. However, only a few studies specifically evaluate the effectiveness of compression therapy without the inclusion of Manual Lymphatic Drainage [21,22]. Given that the use of Manual Lymphatic Drainage is not widely recommended as a standard treatment for lipedema, research highlighting the effectiveness of compression therapy becomes particularly important [22]. The impact of compression therapy is primarily aimed at reducing symptoms such as pain in the affected areas and the overall severity of lipedema symptoms [23].

The surgical treatment for lipedema is liposuction [19,24,25]. It is the only method proven to significantly reduce the volume of adipose tissue in lipedema. Currently applied surgical methods reduce the risk of iatrogenic damage of lymphatics [26]. While liposuction is effective for volume reduction, it is costly and may not be accessible to many patients [25,27]. Additionally, some patients may still need to use conservative treatments post-procedure to prevent the recurrence of lipedema symptoms [28].

Since lipedema is still not fully recognized and misconceptions about the disease persist, it is crucial to use appropriate, precise, and repeatable tools to monitor the effectiveness of lipedema treatment and determine the most suitable approach [29]. Currently, there are no specific guidelines defining the most accurate tools for objectively monitoring treatment effects in lipedema. However, widely used instruments include circumference and volume measurements, quality of life questionnaires, ultrasonography, weight and body composition analysis, Body Mass Index (BMI), and functional scales such as the 6-min walk test [21,29,30].

The aim of the study is to compare the effectiveness of two methods of non-surgical lipedema treatment: compression combined with exercises to exercises only. Moreover, the objective is to present the usage of various qualitative outcome measurements such as 3D scanners, body composition analysis, quality of life assessment, and symptom severity evaluation as tools to determine the results of lipedema conservative therapy.

## 2. Materials and Methods

### 2.1. Patients

**Figure 1:**
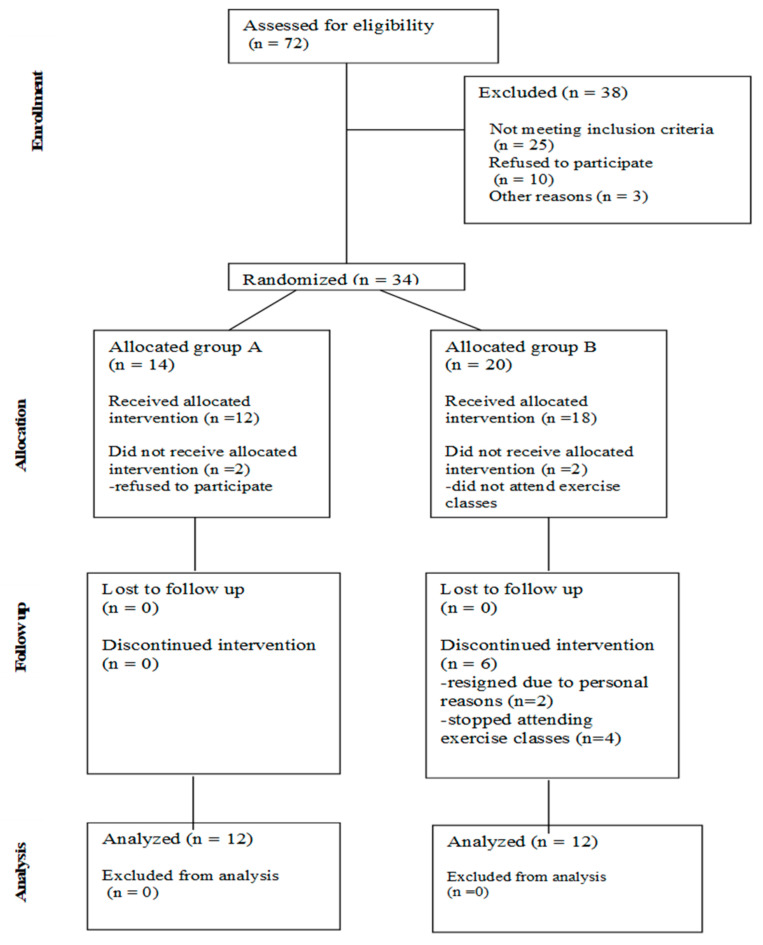
Flow chart presenting the qualification process.

Participants were recruited from an outpatient clinic in Poland and from a social media group for women. The study obtained ethical approval from the Medical University of Gdansk Bioethics Committee (Approval number 912/2021-2022). The recruitment and qualification process took place from February 2023 to September 2023. The study began in September 2023 and ended at the beginning of November 2023. All participants provided written informed consent before being qualified for the study. The inclusion criteria were: female, age between 18 and 60 years, BMI above 25 kg/m^2^, no contraindications for physical activity or compression therapy, currently not pregnant, and presence of lipedema symptoms. The exclusion criteria were: male, age below 18 or above 60 years, BMI below 25 kg/m^2^, orthopedic trauma/surgery within the past 2 years, cancerous disease within the last 5 years, or complications from oncological treatment such as secondary lymphedema, pregnancy, lack of lipedema symptoms, venous thrombosis, exacerbation of chronic diseases, and contraindications for compression or physical activity. Women who expressed a willingness to participate in the study and met the age requirements were invited for eligibility assessments. The evaluation included a medical history focusing on lipedema-specific features, dietary habits, lifestyle, level of physical activity, previous diagnoses, and possible contraindications for compression therapy and physical activity. Additionally, patients underwent a visual evaluation and palpation, which included observation of body proportions, tissue deposition, tissue tenderness and pain, skin condition, consistency of adipose tissue, bruising, and any signs of edema such as the Stemmer sign and pitting tests. Patients who met the inclusion criteria were included in the study. Figure 1 presents a flow chart explaining the qualification process.

### 2.2. Evaluation

Primary evaluation consisted of tape measurement of the smallest circumference of the waist and the largest circumference of the hips, body mass, and height, which were used to calculate the Body Mass Index and Waist to Height ratio. Measurement of treatment effects was made using a 3D scanner Vialux 3D (ViALUX Messtechnik + Bildverarbeitung GmbH, Chemnitz, Germany), body composition analysis using Tanita BC-545N (Tanita Corporation, Tokyo, Japan), quality of life questionnaire (SF-36), and symptom severity survey. The evaluation was made before the intervention and after 8 weeks (post-intervention).

**Figure 2:**
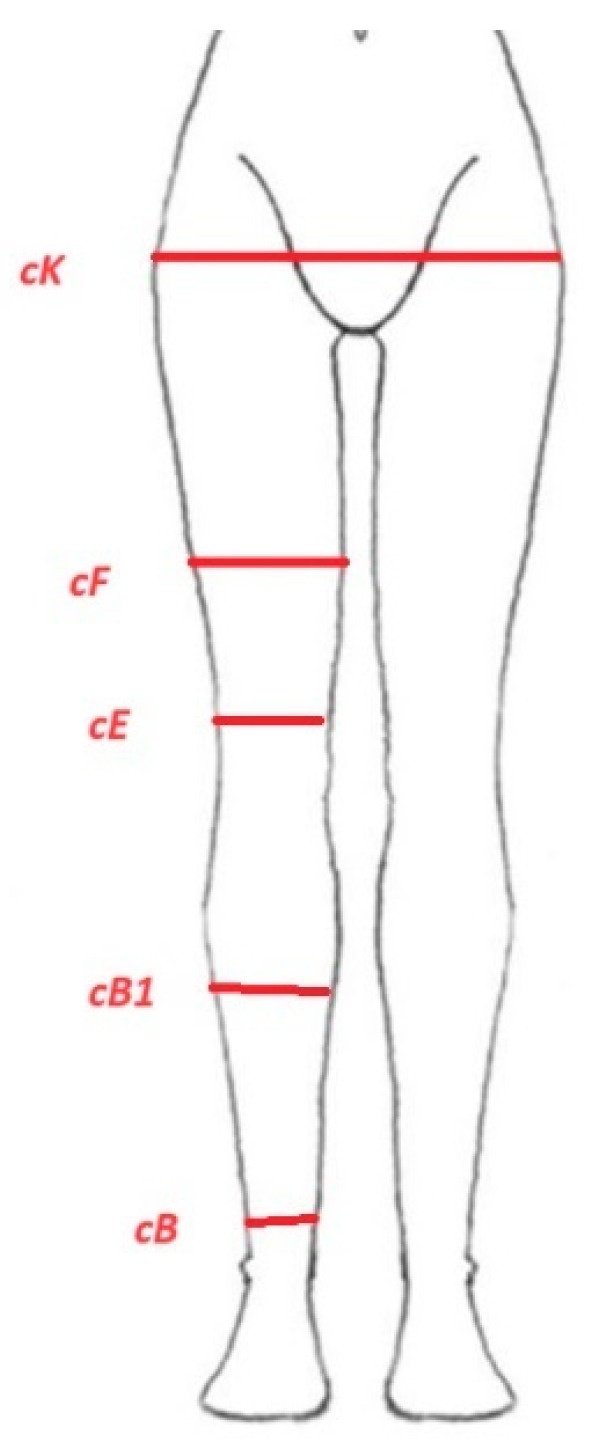
Circumference measurement points using 3D scanner.

Scanner Vialux 3D was used to measure the circumference of the legs and trunk objectively, excluding possible human error during tape measurement. This scanner is an approved class 1 medical device. During the scanning process, the subject stands on a rotating platform with a handrail for support. The platform rotates 360°, capturing over 36 images of the patient, which are then combined to create a three-dimensional body model. The complete model is visualized as a 3D view. For the purpose of the study measurement-5 circumference measures were taken into consideration: cK-hips, cF-½ of the thigh, cE-above the knee, cB1-½ of the calf, and cB-above the ankle (Figure 2). Moreover, the volume of the lower extremities was recorded.

Body composition was evaluated using the Tanita BC-545N with bioelectrical impedance analysis. Parameters measured included Percentage of Fluid, Total Percentage of Fat, Trunk Percentage of Fat Tissue, Right Leg Percentage of Fat, Left Leg Percentage of Fat, Muscle Mass (kg), Trunk Muscle Mass, Right Leg Muscle Mass, and Left Leg Muscle Mass. It was required to wear underwear or a swimsuit during the analysis.

The evaluation of the quality of life was made using the SF-36 questionnaire, which consists of eight dimensions: Physical Functioning, Role Limitations Due to Physical Health, Role Limitations Due to Emotional Problems, Energy/Fatigue, Emotional Well-being, Social Functioning, Pain, and General Health. The rating scale ranged from 0 (worst) to 100 (best) in each of the above-mentioned dimensions.

Symptom severity survey covered the following aspects: pain, sensitivity to touch, heaviness in the extremities, bruising, perceived disproportion between trunk and hips, accumulation of fatty tissue around the hips and thighs, swelling in the lower limbs, and the impact of lipedema symptoms on daily activities. Patients were rating the intensity of each symptom on a scale from 0 to 10, a score of 0 signified no presence of the feature, while a score of 10 indicated the highest level of intensity.

### 2.3. Intervention

Qualified participants were randomly assigned to one of two groups: Group NC, which participated in an exercise program without compression, and Group C, which combined the exercise program with compression therapy. Participants in Group C received compression leggings tailored to each patient based on 3D scanner measurements to ensure accurate sizing. The applied compression was Class 2, seamless, and flat-knitted. Women were instructed to wear the compression garments for as long as possible each day and to record the duration of wear daily.

All of the participants from both groups underwent an 8-week exercise program, which consisted of three one-hour long training units per week-1 under the supervision of a physiotherapist and two independently at home, based on given instructions, and specific exercises. All patients, regardless of the group they were assigned to, followed the same exercise program, exercising three times per week for one hour. Each week patients received specific exercises to perform. The program consisted of breathing exercises, aerobic exercises, strength, stability, and flexibility exercises. Exercise protocols are available upon request from the corresponding author. Patients were asked to record their home training sessions, including the duration, and intensity.

### 2.4. Statistics

**Figure 3:**
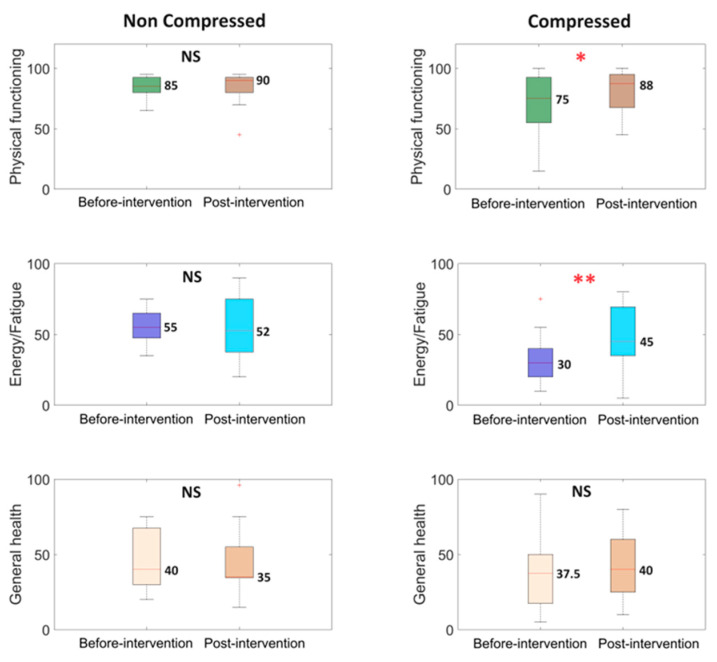
Boxplots illustrate the analysis results for quality of life parameters. The median value is placed next to each boxplot. The two box colors correspond to two different stages of the experiment: ‘Before-intervention’ at the beginning and ‘Post-intervention’ after the experiment. The first column represents the non-compressed case, while the second column represents the compressed case. The ‘*p*’ values were estimated using the Wilcoxon signed-rank test, with * *p* < 0.05, ** *p* < 0.01 and NS—non-statistical, ^+^ indicates outliers.

**Figure 4:**
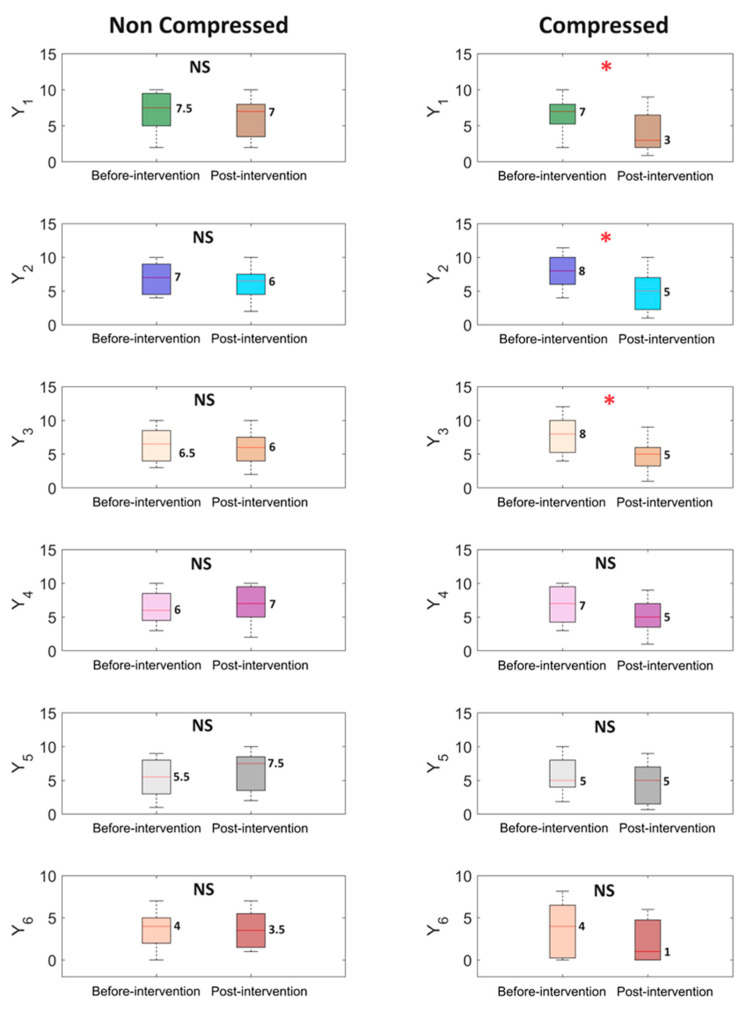
Boxplots illustrate the analysis results for symptom parameters, where: Y_1_ is a subjective level of disproportion, Y_2_ is a subjective level of swelling in lower limbs, Y_3_ is a subjective level of heaviness in extremities, Y_4_ is a subjective level of intensity of bruising, Y_5_ is a subjective level of sensitivity to touch and Y_6_ is the impact of lipedema symptoms on daily activities. The median value is placed next to each boxplot. The two box colors correspond to two different stages of the experiment: ‘Before-intervention’ at the beginning and ‘Post-intervention’ after the experiment. The first column represents the non-compressed case, while the second column represents the compressed case. The ‘*p*’ values were estimated using the Wilcoxon signed-rank test, with * *p* < 0.05, and NS—non-statistical.

**Figure 5:**
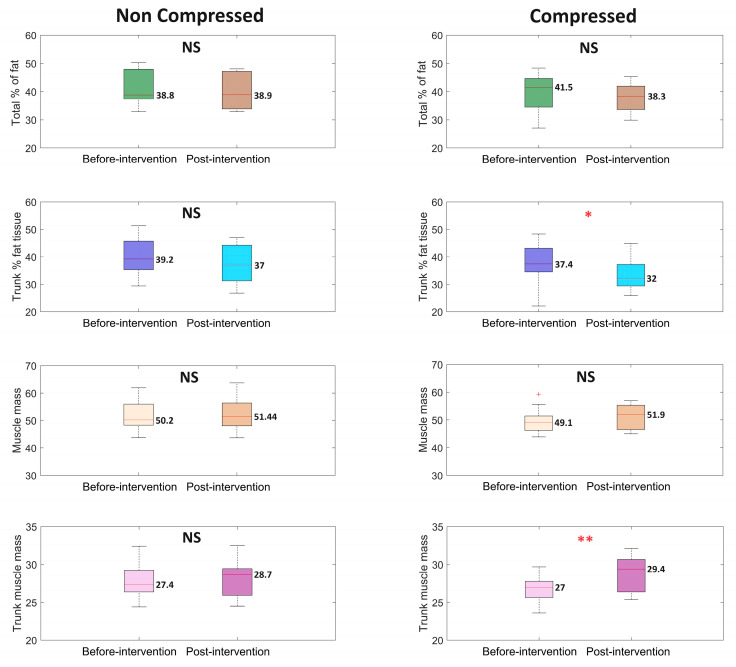
Boxplots illustrate the analysis results for different body composition parameters. The median value is placed next to each boxplot. The two box colors correspond to two different stages of the experiment: ‘Before-intervention’ at the beginning and ‘Post-intervention’ after the experiment. The first column represents the non-compressed case, while the second column represents the compressed case. The ‘*p*’ values were estimated using the Wilcoxon signed-rank test, with * *p* < 0.05, ** *p* < 0.01, and NS—non-statistical, ^+^ indicates outliers.

**Figure 6:**
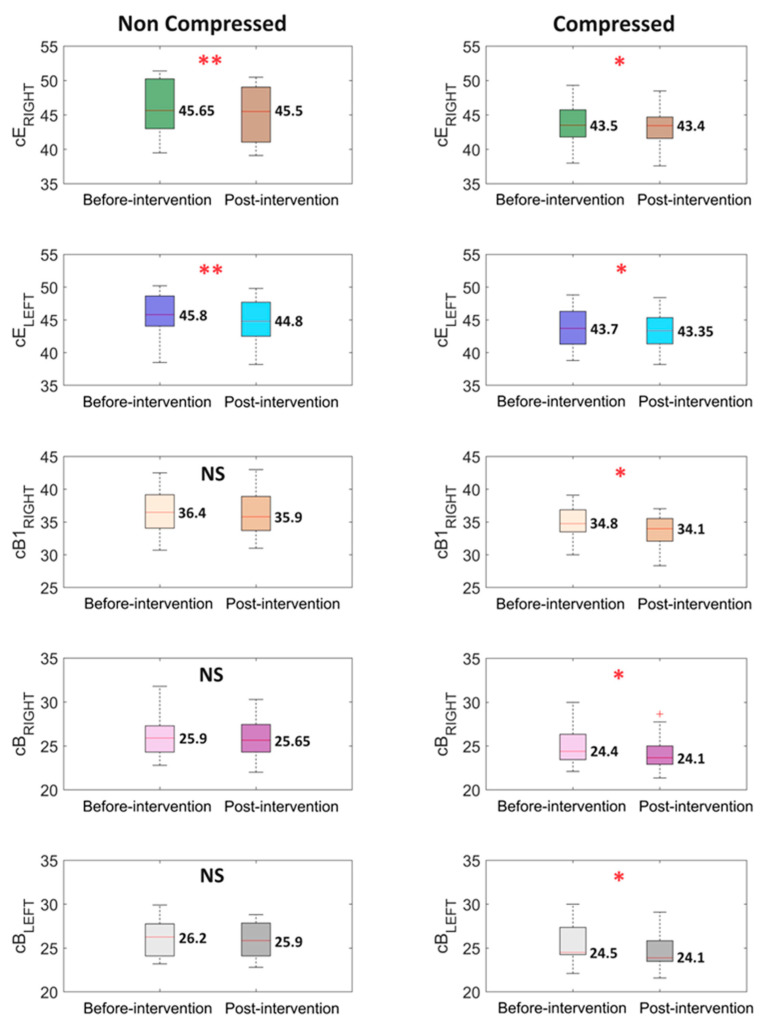
Boxplots illustrate the analysis results for the leg circuits at specific heights. The median value is placed next to each boxplot. The two box colors correspond to two different stages of the experiment: ‘Before-intervention’ at the beginning and ‘Post-intervention’ after the experiment. The first column represents the non-compressed case, while the second column represents the compressed case. The ‘*p*’ values were estimated using the Wilcoxon signed-rank test, with * *p* < 0.05, ** *p* < 0.01 and NS—non-statistical, ^+^ indicates outliers.

To present the results of the analysis, the median along with the first (Q1) and third (Q3) quartiles (as shown in Table 1 and all figures) were used because the data do not follow a normal distribution. Additionally, nonparametric statistical tests were employed for all comparisons. Specifically, the Wilcoxon signed-rank test was used to assess whether the medians of two different stages of the experiment—‘Before-intervention’ at the beginning and ‘Post-intervention’ after the experiment—differed significantly. The results of these analyses are presented in Figures 3–6. All statistical analyses and figure plotting were conducted using MATLAB software (Matlab R2022a 64bit).

## 3. Results

### 3.1. Participants

A total number of 24 lipedema patients were included in the study. Participants were randomly allocated to groups-Group C-compression therapy combined with exercises (*n* = 12), and group NC-exercise program only (*n* = 12). No statistically significant differences were found between groups C and NC, regarding the characteristics of participants. Table 1 presents the characteristics of the patients in total and within groups.

### 3.2. Quality of Life

The results of quality of life measurement using SF-36 pre- and post-intervention are presented in Figure 3.

Significant improvements were observed in patients who underwent compression therapy combined with exercise in two dimensions: Physical Functioning and Energy/Fatigue. Physical Functioning increased in participants from both groups, but only the group that received compression therapy showed statistically significant results. The Energy/Fatigue parameter decreased from 55/100 to 52/100 in the group without compression, while it increased significantly from 30/100 to 45/100 in the compression group. These results are illustrated in Figure 3. General health increased in the group with compression therapy, while it decreased among patients who did not receive compression. The results of the remaining SF-36 dimensions were not statistically significant.

### 3.3. Symptom Severity

The severity of lipedema symptoms before and after the intervention in groups C and NC is presented in Figure 4. A statistically significant reduction was observed in participants from group C in terms of subjective levels of disproportion, heaviness in the extremities, and swelling. The remaining results (subjective impact on daily functioning, level of accumulation of fatty tissue around hips/thighs, level of intensity of bruising, level of sensitivity to touch, and pain) were not statistically significant. The level of experienced pain increased from 4.5/10 to 5/10 in group NC, whereas it decreased from 5/10 to 4/10 in group C. A similar trend was observed in sensitivity to touch, which decreased from 5 to 4.5/10 in group C, while it increased from 5.5 to 7.5/10 in group NC. The subjective level of intensity of bruising decreased from 7/10 to 5/10 in the compression group, while it increased from 6/10 to 7/10 in the non-compression group.

### 3.4. Body Composition

The results of the body composition assessment are presented in Figure 5. The total percentage of fat tissue and total muscle mass did not change significantly among participants from both groups, however significant reduction of the percent of fat tissue in the trunk area and an increase of trunk muscle mass was observed in group C.

### 3.5. Circumference and Volume Using 3D Scanner

The volumes of the right and left lower extremities did not change significantly in either group C or NC. Similarly, there was no significant change in the circumference measurements of the hips (cK) and the midpoint of the thigh (cF). At the midpoint of the calf, the non-significant changes post-treatment could be observed on the right extremity in a non-compression group, and on the left extremity in the compression group. Statistically significant changes were observed in the measurements above the knee (cE) on both the right and left sides in both groups, at the midpoint of the calf (cB1) on the right side in group C and on the left side in group NC, and above the ankle (cB) on both the left and right sides only in group C (Figure 6).

## 4. Discussion

The treatment and management of lipedema symptoms remain challenging due to numerous misconceptions about the condition [4,5,16,31]. The painful and debilitating symptoms experienced by lipedema patients negatively affect their ability to perform daily activities, sustain a fulfilling social life, and diminish their overall quality of life [23,32]. Conservative management of lipedema primarily aims to reduce the intensity of symptoms, alleviate spontaneous and pressure-related pain, decrease the feeling of heaviness in the limbs, and improve quality of life. Compression therapy has been recommended as an effective method for alleviating symptoms and pain associated with lipedema in the recently published German guidelines [23]. It is crucial to highlight that the purpose of compression therapy is not to reduce the volume of adipose tissue, but rather, to relieve the symptoms associated with lipedema [23]. Despite increasing interest in lipedema research, the number of studies presenting the effectiveness of compression therapy without combining it with Manual Lymphatic Drainage is scarce. This highlights the need to emphasize the importance of assessing the effectiveness of compression therapy on its own.

The experience with compression therapy among lipedema patients was evaluated by Paling I. Out of 279 lipedema patients, 54% reported symptom relief from wearing compression garments. Moreover, a correlation between the frequency of wearing compression, and its effectiveness was found [32]. Patients that reported wearing compression every day or 5–6 days a week experienced a greater reduction of lipedema symptoms compared to those that used compression less frequently [32].

The severity of symptoms was previously evaluated in a study by L. Ricolfi, involving 29 women with lipedema who participated in a four-week intervention combining compression therapy with exercise [21]. After undergoing 4 weeks of compression combined with exercises women experienced significant improvement in the severity of lipedema symptoms-spontaneous pain decreased from 3.1/5 to 1.21/5, feeling of swelling in the lower limbs from 3.97/5 to 1.66/5, heaviness in the lower extremities from 3.86/5 to 1.52, hypersensitivity to touch from 2.76 to 1.1/5, and easy bruising from 2.74 to 1.38. All of the improvements were statistically significant [21]. In our study, significant improvements were observed in the group receiving compression therapy: the subjective level of disproportion decreased from 6.5/10 to 3.5/10, the level of heaviness in the extremities lowered from 7.5/10 to 4.5/10, the level of swelling decreased from 7.5/10 to 4.5/10, and bruising decreased from 7/10 to 5/10. The differences in the exercise-only group were not significant; however, it is worth noting that the subjective level of sensitivity to touch increased among these patients (baseline 5.5/10, follow-up 7.5/10), while it decreased among participants in the compression group (baseline 5/10, follow-up 4.5/10).

Unfortunately, there are limited studies that solely assess the effects of compression therapy. However, some research does explore the combination of compression therapy with other components of Complete Decongestive Therapy (CDT). A study by T. Atan assessed the effectiveness of an exercise program combined with Complete Decongestive Therapy and Intermitted Pneumatic Compression among lipedema patients. In this study, the quality of life improved significantly in nearly all SF-36 dimensions (excluding role limitations due to emotional problems) among participants who underwent Complete Decongestive Therapy combined with exercises [30]. The group that received intermittent pneumatic compression and the group that only performed exercises showed significant improvement in Physical Functioning, Social Functioning, and Pain. Additionally, the exercise-only group showed improvement in emotional well-being [30].

Another study by T. Wright assessed the effects of pneumatic compression on the quality of life in 26 lipedema patients [33]. One group underwent pneumatic compression combined with compression therapy, and the other received compression therapy alone. The results indicated significant improvement only in the SF-36 Pain dimension among participants who received both compression and pneumatic compression. Although the remaining results were not statistically significant, improvements were observed in Physical Functioning, General Health, and Energy/Fatigue dimensions after conservative treatment [33].

In our previous pilot study, we assessed the effectiveness of combining compression therapy with exercises versus exercises alone in a smaller patient group (*n* = 6) [22]. The results showed significant improvements in quality of life for participants undergoing compression therapy, specifically in the areas of Physical Functioning, Energy/Fatigue, and Social Functioning. These findings are consistent with our current study, which also demonstrated significant quality of life improvements in the Physical Functioning and Energy/Fatigue dimensions for participants receiving compression therapy combined with exercises, while no significant difference was observed in those performing exercises only. Although the remaining dimensions did not show statistically significant differences, greater improvement was noted in the compression group compared to the non-compression group.

A 3D scanner has not yet been used to monitor treatment effectiveness in lipedema, though circumference measurements have been described by researchers. In a study by M. Volkan-Yazici, circumference measurements were taken before and after a treatment protocol consisting of complete decongestive therapy and intermittent pneumatic compression in 23 lipedema patients using a Perometer [34]. Significant Post-intervention reductions in circumference were observed: at ½ thigh, a reduction of 1.2 cm on the right and 2.8 cm on the left extremity; at mid-patella, a reduction of 0.6 cm on the right and 0.2 cm on the left side; at the smallest circumference below the knee, a reduction of 0.1 cm on the right and 0.7 cm on the left side; and at the largest circumference of the calf, a reduction of 1.4 cm on the right and 1 cm on the left limb [34]. The reduction of circumference could be observed among our study participants who underwent compression combined with exercises and exercises only. The reduction was higher in the group that did not receive compression in one parameter (cE right and left), however in other parameters (cB1 right, cB right and left) the reduction of circumference was significant in the group that underwent compression therapy, while it was not significant in the non-compression group.

Quality-of-life questionnaires and symptom severity surveys, even though self-administered, provide quantitative insights into treatment efficacy, symptom improvement, and daily functioning [21,33]. Although the changes in circumference after the intervention were minimal in both groups—with and without compression therapy—this does not suggest that compression therapy is ineffective. The reduction of circumference in lipedema patients is a gradual process that can be influenced by various external factors, such as the menstrual cycle, warm weather, and hormonal imbalances. Additionally, dietary changes can also affect circumference.

Despite our efforts to minimize bias, this study has some limitations. Although patients were instructed to maintain their usual dietary habits throughout the study, even small changes could have influenced the circumference measurements. Additionally, we believe extending the observation period beyond eight weeks and incorporating a longer-term follow-up, such as one year, would provide valuable insights into the durability of the results. Furthermore, future research would benefit from conducting the study with a larger sample size.

## 5. Conclusions

Lipedema is a complex disease that is still challenging to manage. Compression therapy combined with exercises is an effective conservative treatment method for lipedema patients. Using a combination of compression therapy and physical activity has a positive impact on alleviating lipedema symptoms and increasing quality of life. Quality of life and symptom severity assessment are accurate tools for determining treatment effectiveness.

There was an improvement in circumference at some of the measured points in both the group that underwent compression therapy combined with exercises and the group that performed exercises only. Therefore, using solely circumference measurement may not be fully appropriate for assessing the effectiveness of compression therapy, as compression therapy is not primarily intended to reduce circumference in lipedema patients, but rather to alleviate pain and reduce lipedema symptoms.

## Figures and Tables

**Table 1:** Characteristics of patients qualified for the study. Median values, first quartile Q1 (lower index) and third quartile Q3 (upper index) are presented in the table regarding age, BMI, WHtR, and body weight. NS—non-statistically significant.

Feature	Total (*n* = 24)	Group C (*n* = 12)	Group NC (*n* = 12)	*p*
Age, years (median$\frac{Q3}{Q1}$)	$35.5_{30.75}^{46}$	$31.5_{28.5}^{42}$	$37.5_{33.75}^{47}$	NS
BMI (median$\frac{Q3}{Q1}$)	$33.4_{27.9}^{37.07}$	$32.4_{27.8}^{34.5}$	$35.15_{29.4}^{39.2}$	NS
WHtR (median$\frac{Q3}{Q1}$)	$0.54_{0.49}^{0.58}$	$0.53_{0.49}^{0.58}$	$0.55_{0.51}^{0.59}$	NS
Body weight (kg) (median$\frac{Q3}{Q1}$)	$89.3_{80.35}^{104.17}$	$89.05_{74.4}^{94}$	$96.55_{81.5}^{107}$	NS
Previous lipedema diagnosis				
Yes	1	1	0	
No	23	11	12	
Lipedema symptoms				
Heaviness in lower extremities	24	12	12	
Pain at palpation	24	12	12	
Spontaneous pain in lower/upper extremities	18	10	8	
Disproportion between slimmer trunk and enlarged limbs	22	11	11	
Easy bruising	22	12	10	
Accumulation of fat tissue mostly around legs	22	12	10	
Difficulties losing weight in the affected areas	24	12	12	
Swelling around the ankles depending on temperature	20	10	10	
Lipedema Stage				
Stage 1	6	3	3	
Stage 2	12	8	4	
Stage 3	6	1	5	
Family history				
Yes	20	9	11	
No	4	3	1	
Onset				
Puberty	21	10	11	
Pregnancy	3	2	1	
Menopause	0	0	0	
Other	0	0	0	
Declared level of physical activity				
None	4	2	2	
Low	6	4	2	
Medium	12	6	6	
High	2	0	2	

## Data Availability

The datasets used and/or analyzed during the current study are available from the corresponding author upon reasonable request, due to confidential patient information included in the database.
